# A new insight into ayahuasca’s adverse effects: Reanalysis and perspectives on its mediating role in mental health from the Global Ayahuasca Survey (GAS)

**DOI:** 10.1371/journal.pmen.0000097

**Published:** 2025-04-30

**Authors:** Óscar Andión, José Carlos Bouso, Jerome J. Sarris, Luís Fernando Tófoli, Emérita Satiro Opaleye, Daniel Perkins

**Affiliations:** 1 Research Sherpas, Mieres, Girona, Spain; 2 International Center for Ethnobotanical Education, Research and Services (ICEERS), Barcelona, Spain; 3 Medical Anthropology Research Center, Department of Anthropology, Philosophy and Social Work, University of Rovira I Virgili, Tarragona, Spain; 4 Psychae Institute, Melbourne, Australia; 5 NICM Health Research Institute, Western Sydney University, Westmead, New South Wales, Australia; 6 Centre for Mental Health, Swinburne University, Melbourne, Victoria, Australia; 7 The Florey Institute of Neuroscience and Mental Health and The Department of Psychiatry, Melbourne University, Melbourne, Australia; 8 Interdisciplinary Cooperation for Ayahuasca Research and Outreach (ICARO), School of Medical Sciences, University of Campinas, Campinas, Brazil; 9 Department of Psychobiology, Universidade Federal de São Paulo, São Paulo, Brazil; 10 School of Population and Global Health, University of Melbourne, Melbourne, Australia; 11 Centre for Mental Health, Swinburne University, Melbourne, Australia; Magna Graecia University of Catanzaro: Universita degli Studi Magna Graecia di Catanzaro, ITALY

## Abstract

Ayahuasca is a decoction native to the Amazon, where it plays a central role in the traditional medicine of many local cultures and has expanded internationally over the last decades. Ayahuasca has also attracted the interest of scientists for its potential benefits on mental health, but its adverse effects are under-researched. We analyzed data from the Global Ayahuasca Survey, including 10,836 participants who rated predetermined adverse effects. Data were collected from March 1st, 2017, to December 31st, 2019, and accessed for analysis on November 30th, 2021. Only DP and JJS had access to identifiable participant data. Machine learning and statistical methods were used to examine the relationship between sample characteristics, post-ayahuasca adverse mental states, and mental health outcomes measured by the 12-Item Short Form Survey (SF-12). Among participants, 14.2% (767) had a prior anxiety disorder and 19.7% (1,064) a depressive disorder. Despite this, the median SF-12 score was 50.16, comparable to the general population. A history of anxiety or depression was associated with more adverse mental states after ayahuasca use. However, increased experiences of “visual distortions” and higher ayahuasca use correlated with better mental health. Women reported more adverse states but did not show worsened mental health. The classification of adverse mental states in psychedelic research should be reconsidered, as certain experiences traditionally labeled as negative may contribute to long-term psychological benefits. The context in which these experiences occur, along with individual factors, plays a crucial role in determining whether these states lead to positive or negative outcomes. Understanding these dynamics is essential for improving harm reduction strategies and maximizing therapeutic potential. Individuals with a history of depression require special attention, as they are more prone to experiencing post-ayahuasca adverse mental states and may benefit from additional psychological support.

## Introduction

Ayahuasca is a decoction native to the Amazon, where it plays a central role in the traditional medicine of many local cultures, likely for millennia [1]. Ayahuasca is also the sacrament of some syncretic Brazilian religions, some of which have expanded internationally across all continents over the last four decades [2]. Ayahuasca itself has undergone a more recent process of globalization [3] to the point where both psychiatrists and priests have made respective calls to their colleagues to be more informed [4,5]. At the same time, scientific production of ayahuasca research is also exponentially increasing, although this mostly focuses on its potential benefits for mental health and less on its potential risks and adverse effects [6].

Ayahuasca is both the Quichua name for the vine *Banisteriopsis caapi* and for any decoction made with it. *B. caapi* contains harmala alkaloids (mainly harmine, harmaline, and tetrahydroharmine), which are MAOI inhibitors in the gastrointestinal tract [7]. The most common plants mixed with *B. caapi* are the leaves of the bush *Psychotria viridis* or the leaves of the vine *Diplopterys cabrerana*, both containing the amine N,N-Dimethyltryptamine (DMT). DMT is a partial agonist of serotonin receptors, mainly 5-HT2A and 5-HT1A, and is responsible for the hallucinatory effects of ayahuasca [8]. When administered in a laboratory, ayahuasca slightly increases heart rate and blood pressure [9,10], transiently increases prolactin and cortisol, and modifies immunological markers [11,12]. It induces decreases in EEG power in the delta, theta, and alpha frequency bands [12–14], reduces top-down control, and increases bottom-up information transfer in the brain [15]. It increases blood perfusion in the frontal and paralimbic brain regions in both normal [16] and depressed subjects [17], and decreases the activity of the default mode network [18].

In terms of subjective effects, ayahuasca increases dose-dependent responses compared to placebo on visual analog scales of “any effect”, “good effects”, “liking”, “visions”, “stimulated”, and “high”, without altering “drunkenness”. It increases the subscales of Affect, Cognition, Somaesthesia, Perception, Intensity but not Volition of the Hallucinogen Rating Scale (HRS), and the measures of stimulatory effects (A scale), euphoria (MBG scale), and somatic symptoms (LSD scale) from the Addiction Research Center Inventory (ARCI) questionnaire [9–12]. This profile of subjective effects has also been observed in naturalistic settings, alongside the induction of mystical experiences assessed with the Mystical Experience Questionnaire (MEQ) [19].

Contemporary clinical research has studied the effects of ayahuasca on depression [19–22], anxiety disorders [23,24], grief [25–27], emotional regulation [28], drug dependence [29], general wellbeing [30,31], quality of life, and general mental health [32], and even creativity [33]. Studies using health indicators employed by governments to assess the general health of their populations have found improved results in such indicators as healthy daily habits, use of psychiatric drugs, cholesterol, and other imbalances caused by lifestyle in large samples of regular ayahuasca users in both Spain and the Netherlands [34,35]. Regular long-term use of ayahuasca has been associated with slight improvements in neuropsychological functions [36–39] and neuroplasticity [38,40].

Despite the scientific interest and increasing number of publications regarding the effects of ayahuasca in both clinical and naturalistic settings, little research has been conducted on its potential acute and subacute adverse effects, as well as its possible consequences for health and/or mental health. Recently, we published a report in which we reviewed all the scientific knowledge reporting ayahuasca’s adverse effects, and where we also reported the adverse effects collected in the Global Ayahuasca Survey (GAS), a survey conducted on more than 10,000 subjects who attended an ayahuasca ceremony, comprising participants from more than 50 countries [6]. In that paper, we reported a high prevalence (55.4%) of adverse mental sates after ayahuasca use, with 12% seeking professional support for these effects, but also a low frequency of severe post-use adverse states (4.4%). Moreover, more post-use adverse states were reported for participants with higher ayahuasca uses, with previous mental health disorders, and in non-traditional contexts of ayahuasca use. However, our previous study did not analyze the relationship of the adverse mental states with the participants’ current mental health. Moreover, previous studies analyzing the relationship of ayahuasca’s adverse effects with participants’ health status seem to show contradictory results. While acute adverse effects have been related to psychiatric condition improvement [41], the number of adverse effects after ayahuasca use was negatively associated with current mental health and perceived improvement in psychological wellbeing in another study [42]. Moreover, subjective experiences typically experienced by ayahuasca users, such as visual distortions or hallucinations, could be perceived as beneficial rather than adverse effects. In this context, It seems necessary to explore the mediating variables involved in the development of potential harms or benefits following an adverse event.

In this new analysis, we aim to extend our previous study results by trying to answer the question raised above. As noted, post-use adverse mental effects are related to independent variables (e.g., sociodemographic, clinical, or history of ayahuasca use variables) that are also expected to be related to participants’ current mental health status. Thus, we hypothesize a mediational relationship where we will test the relationships of the independent variables on reported adverse states increment after ayahuasca use and the relationships of these variables (the independent variables and adverse effects) on ayahuasca users’ current mental health. Moreover, in this study, we include as independent variables acute ayahuasca experiences, the spiritual significance, and the extreme fear experienced during the ceremonies. The first has been associated with psychedelic experiences [43,44] and an increased likelihood of experiencing adverse effects [6]. The second has been related to extended difficulties following psychedelic drugs use [45] and has been linked with “bad trips” [46]. As explanatory models can be difficult to interpret when a high number of exogenous and mediation variables are included in the analysis, in this study, a machine learning and classical statistical combined approach is used [47,48] to better analyze our aims.

## Methods

### Ethics statement

The study was approved by the University of Melbourne Human Research Ethics Committee (HREC number 1545143.3), and all participants provided their informed consent via an initial question in the online survey. Participants were unable to proceed if consent was not provided.

### Sample

The study sample was drawn from 10,836 participants of an online survey conducted between 2017 and 2019. The survey was available in six languages (English, Portuguese, Spanish, German, Italian, and Czech) and collected data from participants in more than 50 countries. All participants were over 18 years old and had used ayahuasca on one or more occasions.

Survey participation was promoted through websites and email invitations from relevant organizations, ayahuasca retreat centers, ayahuasca churches, online groups and forums, via Facebook, and flyers at conferences and events. No financial incentives were offered. Data was cross-checked to remove suspected duplicate responses, and information from partially completed surveys was preserved. Given the opaque nature of the ayahuasca-using population in many countries (where this practice is either prohibited or where its legal status remains unclear) a non-random sampling method was chosen.

After missingness was explored, only participants without missing responses in any of the study variables were included in the analyses (5,400; 49.8%; see Statistical Analysis section). No significant differences were observed between included and excluded participants in terms of sex distribution, context of ayahuasca use, age at first ayahuasca use, frequency of ayahuasca uses in the last year, lifetime ayahuasca uses, current physical or mental health scores, and prevalence of lifetime anxiety or alcohol use disorders (all *ps* ≥.082). However, participants included in the analyses were older [40.94 (12.01) *vs.* 39.63 (12.39); t_(10,687)_ = 5.54; *p* <.001], had higher educational levels [university education: 65.2% (3,522) *vs.* 58.6% (3,133); χ^2^_(1)_ = 49.23; *p* <.001] (To reduce the comparison of education levels between included and excluded participants, previous to the analyses the between groups difference in the variable, it was recoded to a dichotomic variable measuring university studies.), and reported fewer ayahuasca mental adverse effects [2.70 (4.26) *vs.* 2.95 (4.55); t_(7,833)_ = 2.28; *p* =.023} than the excluded participants. Finally, a lower frequency of lifetime depression [19.7% (1,064) *vs.* 21.6% (516); χ^2^_(1)_ = 3.74; *p* =.053] and substance use disorders [9.2% (496) *vs.* 12.4% (296); χ^2^_(1)_ = 18.73; *p* <.001] was observed among the included participants compared to those excluded.

### Measures

Demographic information such as age, sex, education, and country of residence was obtained from participants, in addition to their lifetime history of mental health diagnoses and detailed ayahuasca drinking history, including frequency, patterns, and contexts of use [42]. A single item question was used to measure the extent to which extreme fear or panic was experienced during the ayahuasca session, scored from 0 “Not at all” to 10 “Very much.” The degree of spiritual significance of ceremonies was reported using the spirituality component of the Persisting Effects Questionnaire [49] that involved a six-point scale (ranging from not at all significant to the single most spiritually significant experience of my life). The mental component score of the SF-12 instrument was used to evaluate current mental health status in accordance with the guidelines for the SF-12 instrument, with scores ranging from 0 to 100 and higher scores signifying better mental health [50].

Acute adverse mental health effects were assessed using a question about short- to medium-term mental health, emotional, or perceptual changes occurring in the weeks or months after consumption. This was based on the PHQ-4 [51], supplemented with six additional items derived from the ayahuasca literature. The question asked respondents, “In the weeks or months after your ayahuasca ceremonies/sessions, have you ever experienced an increase in any of the following?” Responses were collected using a modified version of the PHQ-4 four-point scale (Not at all, Slightly, Moderately, Very Much). Note that this question did not specifically describe these items as adverse effects.

### Statistical analysis

#### Data analysis strategy.

**Fig 1:**
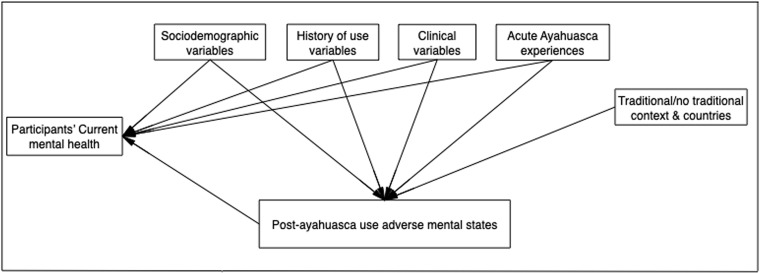
Hypothesized mediational post-ayahuasca use adverse mental states model.

The main objective of the study is to analyze the relationships between participants’ reported adverse mental states after ayahuasca use and their current mental health status using Structural Equation Modeling (SEM). As mentioned above, due to the expected relationships between the study variables, we test a hypothesized mediational model. The mediational model is briefly presented in Fig 1.

Before applying Structural Equation Modeling (SEM) analysis, we employ the Random Forest algorithm to select from the 24 initial predictors those that would improve the R-squared and reduce the root mean square error (RMSE) when predicting participants’ current mental health. This step aims to refine our predictor set, enhancing the clarity of the SEM model by potentially reducing the number of variables involved. Random Forest is a widely used machine learning procedure for feature selection that can enhance the efficiency of models [52,53]. It has been applied in mental health research to similar ends (e.g., [54,55]. Prior to these analyses, participants were selected, data were explored, pre-processed, and a multivariate outlier detection algorithm was employed to finalize the sample to be used. This preparatory phase ensures that the data feeding into both the Random Forest and SEM analyses are optimized for quality and relevance.

#### Sample selection and data pre-processing.

As it is an online survey research and missing response are frequently observed, missingness responses were explored prior to the analyses. As this is an exploratory study and none previous research has specifically analyzed the relationships between clinical, ayahuasca history of use variables and the adverse post-ayahuasca use states with participants’ current mental health, after the data exploration, in this study participants with missing response were deleted. Given that the distribution of variables is a critical factor when using multivariate statistical procedures based on Euclidean distances, such as k-nearest neighbors algorithms and SEM, before proceeding with the analyses, an exploration of the distributions of the included variables was conducted to ensure the appropriateness of the statistical techniques and to facilitate accurate interpretations of the findings.

Lifetime and last year ayahuasca use displayed high non-normality values (Skewness > 8.475 and Kurtosis > 98.80), to reduce excessive kurtosis these variables were transformed to ordered one based on the 10th to 100th percentiles (see Table 1). Additionally, the two categorical variables included in the study were recoded to dichotomous variables: traditional vs. non-traditional countries (Brazil, Ecuador, Peru, Bolivia, Colombia and Venezuela countries of birth vs. other countries), and traditional vs. non-traditional context of use (Ayahuasca Church and Traditional Shaman vs. non-traditional supervision and others contexts of use). A high correlation was observed between lifetime and last year ayahuasca use. However, since no linear dependence was detected between the variables, and one of the random forest algorithms we plan to use in our analysis, Recursive Feature Elimination, has been recommended for variable selection in the context of multiple correlated variables [56], the last year ayahuasca use variable was not deleted.

**Table 1:** Sample description (n = 5,400).

Sociodemographical variables	%	n
Female	47.1	2,546
Age^*^	40.94	12.01
Education		
Primary school or lower	.5	24
Secondary school	1.0	52
Trade, techical, vocational qualification/certificate	7.2	389
Completed high school	10.6	571
Diploma/advanced diploma/assicat’s degree	15.6	841
Undergraduated/bachelor’s degree	30.3	1,634
Master’s degree or post-graduated/diploma	29.7	1,604
PhD/Doctorate	5.3	284
Residence region		
Brazil	46.0	2,485
Other Latin America country	5.3	285
Europe	26.5	1,433
North America	17.1	926
Australia & NZ	4.4	239
Asia and Middle East	.6	32
Traditional countries	48.4	2,613
**Ayahusaca use history variables**		
Age of onset^*^	31.49	12.34
Age of onset before 18 years old	476	8.81
Last year ayahuasca uses		
No use	16.0	865
One use	8.5	459
Two uses	7.1	381
3–4 uses	9.7	522
5–8 uses	8.9	483
9–20 uses	12.1	656
21–30 uses	14.9	806
31–35 uses	2.8	150
36–45 uses	10.1	543
More than 45 uses	9.9	535
Lifetime uses		
1–2 use	13.3	716
3–4 uses	10.6	575
5–7 uses	7.9	424
8–14 uses	8.2	445
15–30 uses	11.2	605
31–75 uses	8.7	471
76–200 uses	12.6	681
201–396 uses	7.5	404
397–700 uses	10.2	552
More than 700 uses	9.8	527
**Context**		
Traditional context	67.4	3,637
Non-traditional context	32.6	1,763
**Mental health**		
Anxiety disorder	14.2	767
Depressive disorder	19.7	1,064
Substance use disorder	9.2	496
Alcohol use disorder	10.0	538
SF-12 mental health^*^	50.16	9.11
Extreme fear^*^	3.62	3.21
Spiritual significance^*^	4.95	1.11
**Mental health post-ayahuasca use adverse mental states**		
Emotional cognitive adverse states^*^	1.72	3.29
Psychotomimetic adverse states^*^	.99	1.68

* : indicated a continuous variable, thus mean and standard desviation are reported.

The studied variables were pre-processed specifically for each statistical test. For k-nearest neighbors multivariate outlier detection algorithms and numerical data (continuous and ordered variables) for SEM were standardized and scaled, respectively. No multivariate outliers were detected. For Random Forest tests, variables were used in their original scale and data were spilt into training dataset (n = 4,320) and test dataset (n = 1,080) for feature selection and model training and for model evaluation respectively. SEM was performed using the total sample, leveraging the full dataset to ensure robust analysis and comprehensive results.

#### Data analyses.

The statistical analyses were performed using R version 4.2.3 [57]. Multivariate outlier detection was conducted using the Natural Neighbor searching algorithm [58] as implemented in the DDoutlier package [59]. Data preprocessing and Random Forest analyses were carried out using the caret package version 6.0-94 [60]. The mediational model was tested using the lavaan package version 0.6-12 [61].

For the Random Forest Recursive Feature Elimination (RFE) variables selection, the number of resamples was set to 50 and bootstrap was set to 1000. Random Forest was performed using repeated k-fold cross-validation, with 10 partitions and 5 repetitions. The mediational model (Fig 1) was tested following the recommendations by Bauer and Curran (2019) for SEM analyses with the lavaan package. The Robust Diagonally Weighted Least Squares (DWLS; [62,63]) estimation method was used to test the models. Fit indices (Chi-square test, RMSEA, TLI, CFI, and SRMR) were used to assess the model’s fit to the data [64,65]. Covariance between exogenous variables was fixed to their sample values.

For a clearer interpretation of the effects studied, both fully standardized and partially standardized estimates were reported for the numerical and dummy variables, respectively [66]. Finally, statistical power analyses for mediational, indirect, direct, and total effects (π) based on Monte Carlo Simulation were conducted using the Webpower package [67]. The number of Monte Carlo simulations and bootstraps were set to 100 and 5000, respectively.

While sociodemographic variables are included in the analyses and tables, the results section primarily focuses on the history of use, clinical, acute experiences, context, and country as exogenous variables.

## Results

The sample characteristics are summarized in Table 1. Approximately half of the participants were female (47.1%; 2,546), with an average age of 40.9 years (SD = 12.01). The majority of the participants (80.9%; 4,363) held an education level higher than a diploma or advanced diploma and were from Brazil (46.0%; 2,485). The mean age at which participants first used ayahuasca was 31.5 years (SD = 12.34). Only 16.0% (865) of the participants had not used ayahuasca during the previous year, while 59.9% (3,236) had used ayahuasca more than 31 times and 20.0% (1,079) had used it more than 397 times in their lifetime. In terms of context, 67.4% (3,637) of the sample used ayahuasca in a traditional setting. Additionally, 14.2% (767) of participants reported a previous anxiety disorder, 19.7% (1,064) a depressive disorder, and the mean mental health score of the sample, assessed with the SF-12, was 50.16 (SD = 9.11).

### Predictors selections: Random forest analyses

From the initial set of 24 variables included in the study, the RFE algorithm selected 23 variables that showed the highest R-squared and the lowest RMSE (.259 and 7.91, respectively). However, it was determined that a set of 20 features could represent an optimal balance, exhibiting an R² of.256 and an RMSE of 7.92. Moreover, the Random Forest regression model with 20 variables, used to predict participants’ current mental health, exhibited R-squared and RMSE values of.282 and 7.794, respectively and revealed that gender had no significant impact on predicting mental health outcomes in this sample (see Table A and B in S1 Text). Consequently, the variables Alcohol use disorder, Drug use disorder, “Not being able to stop or control worrying”, “Nightmares, or disturbing thoughts, feelings, or sensations” and gender were not included in the subsequent analyses.

### Ayahuasca adverse mental effects mediational model

The model fit indices indicated a high fit to data for the hypothesized Ayahuasca adverse mental effect mediational model (χ² = 1.295, p =.523; CFI = 1.00; TLI = 1.00; RMSEA <.001; RMSEA90% CI =.000 -.024, and SRMR =.001). Although the hypothesized mediational model explained nearly 30.0% (R² =.298) of the variance in participants’ current mental health, the explained variance for the ayahuasca adverse effects included in the model ranged from R² =.060 to R² =.206 (“Visual distortions” and “Difficulty knowing what is real and not real”). The relationships between the variables studied are detailed below by sections.

### Exogenous variables relationships with participants’ reported adverse mental states

As observed in Table 2, ayahuasca history of use variables did not show significant relationships with the participants’ reported increases for half of the adverse mental states studied. However, higher participants’ age of onset and lifetime use were related to greater reported increases of “Feeling down, depressed, or hopeless” (β =.091 -.097; *ps* <.037); “Hearing or seeing things that other people do not hear or see” (β =.178 -.103; p <.006), “Visual distortions” (β =.113 -.146; *p* <.001), and “Feeling energetically attacked or a harmful connection with a spirit world” (β =.116 -.226; *p* ≤.001).

**Table 2:** Exogenous variables relationships with reported increases in post-ayahuasca use adverse mental states.

	Z	*p*	*β*	*se*	π
**Feeling nervous, anxious, or on edge**					
Education	.755	.450	-.015	.021	.980
Age	3.20	.001	-.122	.04	.999
Last year uses	1.58	.114	.052	.036	.999
Age of onset	.47	.639	.019	.043	.960
Lifetime uses	.12	.903	.005	.043	.950
Anxiety	4.57	<.001	.245	.058	.999
Depression	1.65	.10	.080	.052	.999
Traditional/no traditional context	2.55	.011	.108	.046	.999
Traditional/no traditional country	.65	.512	-.030	.050	.960
Spiritual significance	4.29	<.001	-.076	.019	.999
Extreme fear	17.47	<.001	.319	.020	.999
**Little interest or pleasure in doing things**					
Education	2.09	.037	-.043	.022	.999
Age	2.52	.012	-.095	.039	.999
Last year uses	.33	.738	.012	.036	.970
Age of onset	1.09	.275	.043	.041	.970
Lifetime uses	.04	.971	.001	.042	.980
Anxiety	1.41	.158	.086	.063	.999
Depression	2.17	.030	.117	.056	.999
Traditional/no traditional context	.59	.558	.027	.048	.990
Traditional/no traditional country	.58	.560	-.029	.051	.940
Spiritual significance	4.03	<.001	-.079	.020	.999
Extreme fear	11.15	<.001	.220	.020	.999
**Feeling down, depressed, or hopeless**					
Education	.92	.36	-.018	.021	.980
Age	3.72	<.001	-.152	.043	.999
Last year uses	1.00	.318	-.033	.035	.999
Age of onset	2.08	.037	.091	.046	.999
Lifetime uses	2.35	.019	.097	.044	.999
Anxiety	1.16	.247	.065	.059	.970
Depression	5.97	<.001	.291	.052	.999
Traditional/no traditional context	2.97	.003	.128	.046	.999
Traditional/no traditional country	1.53	.126	.070	.049	.999
Spiritual significance	4.36	<.001	-.078	.019	.999
Extreme fear	13.10	<.001	.247	.020	.999
**Feeling disconnected or alone**					
Education	2.93	.003	-.052	.019	.999
Age	4.27	<.001	-.140	.036	.999
Last year uses	.84	.402	-.025	.033	.960
Age of onset	1.92	.055	.068	.039	.999
Lifetime uses	.161	.872	.006	.038	.960
Anxiety	1.90	.057	.096	.055	.999
Depression	5.62	<.001	.248	.048	.999
Traditional/no traditional context	4.87	<.001	.192	.043	.999
Traditional/no traditional country	3.12	.002	.135	.047	.999
Spiritual significance	3.08	.002	-.051	.018	.999
Extreme fear	14.93	<.001	.255	.019	.999
**Difficulty knowing what is real and not real**					
Education	2.17	.030	-.042	.022	.999
Age	3.51	<.001	-.134	.043	.999
Last year uses	1.10	.273	-.038	.039	.980
Age of onset	1.12	.263	.046	.042	.990
Lifetime uses	1.16	.248	-.049	.048	.990
Anxiety	1.92	.054	.113	.066	.999
Depression	2.97	.003	.154	.058	.999
Traditional/no traditional context	2.80	.005	.125	.050	.999
Traditional/no traditional country	2.08	.038	.102	.055	.999
Spiritual significance	2.92	.003	.063	.024	.999
Extreme fear	16.62	<.001	.319	.022	.999
**Hearing or seeing things that other people do not hear or see**					
Education	2.88	.004	-.054	.019	.999
Age	4.30	<.001	-.135	.033	.999
Last year uses	1.27	.203	-.041	.034	.999
Age of onset	5.29	<.001	.178	.035	.999
Lifetime uses	2.74	.006	.103	.039	.999
Anxiety	.61	.544	-.035	.061	.970
Depression	2.10	.036	.103	.051	.999
Traditional/no traditional context	3.85	<.001	.161	.042	.999
Traditional/no traditional country	3.75	<.001	-.172	.023	.999
Spiritual significance	3.93	<.001	.085	.023	.999
Extreme fear	10.82	<.001	.200	.019	.999
**Visual distortions**					
Education	2.78	.005	-.048	.018	.999
Age	3.99	<.001	-.117	.030	.999
Last year uses	.345	.730	-.010	.031	.960
Age of onset	3.59	<.001	.113	.032	.999
Lifetime uses	4.14	<.001	.146	.036	.999
Anxiety	.10	.919	.005	.055	.960
Depression	3.18	.001	.146	.047	.999
Traditional/no traditional context	2.70	.007	.108	.041	.999
Traditional/no traditional country	4.43	<.001	-.191	.044	.999
Spiritual significance	4.01	<.001	.079	.020	.999
Extreme fear	8.98	<.001	.155	.018	.999
**Feeling “energetically attacked” or a harmful connection with a “spirit world”**					
Education	1.60	.109	-.032	.021	.990
Age	6.09	<.001	-.190	.034	.999
Last year uses	2.43	.015	-.082	.037	.999
Age of onset	3.27	.001	.116	.038	.999
Lifetime uses	5.99	<.001	.226	.041	.999
Anxiety	1.88	.060	.109	.063	.999
Depression	4.52	<.001	.231	.056	.999
Traditional/no traditional context	4.00	<.001	.176	.048	.999
Traditional/no traditional country	4.34	<.001	-.200	.050	.999
Spiritual significance	1.07	.286	.023	.023	.999
Extreme fear	15.88	<.001	.309	.021	.999

^1^: partially standarized estimates are reported for dichotomical variables.

Related to participants’ previous mental health disorders, anxiety was associated with greater reported increases of “Feeling nervous, anxious, or on edge” (β =.245; *p* <.001) and “Difficulty knowing what is real and not real” (β =.113; *p* =.054). Moreover, anxiety approached significant levels in increasing “Feeling disconnected or alone” (β =.096; *p* =.057) and “Feeling energetically attacked or a harmful connection with a spirit world” (β =.109; *p* =.060). However, a previous diagnosis of depression was associated with greater reported increases of all the studied adverse post-ayahuasca states (β ≥.103; *p* ≤.036), with the sole exception of “Feeling nervous, anxious, or on edge” (β =.080; *p* =.100) (Table 2).

Participants who used ayahuasca in non-traditional contexts reported greater increases of all the adverse states (β ≥.108; *p* ≤.007), with the only exception of “Little interest or pleasure in doing things” (*p* =.558). However, a different pattern of associations was observed for traditional vs. non-traditional countries. Participants from non-traditional countries showed greater increases of “Feeling disconnected or alone” (β =.135; *p* =.002) and “Difficulty knowing what is real and not real” (β =.102; *p* =.038), but they also reported lower increases in “Hearing or seeing things that other people do not hear or see” (β = -.172; *p* <.001), “Visual distortions” (β = -.191; *p* <.001), and “Feeling energetically attacked or a harmful connection with a spirit world” (β = -.200; *p* <.001) (Table 2).

As it was observed for context and country variables, the participants’ reported increases of acute ayahuasca experiences showed a specific pattern of relationships with the post-use adverse mental states. While participants reported the intensity of extreme fear was related to higher increases of all the adverse states (β ≥ -.155; *ps* <.001), participants’ reported intensity of spiritual significance was related to lower increases of “Feeling nervous, anxious, or on edge,” “Little interest or pleasure in doing things,” “Feeling down, depressed, or hopeless,” and “Feeling disconnected or alone” (β = -.051; *p* ≤.002), but with higher intensity of “Difficulty knowing what is real and not real,” “Hearing or seeing things that other people do not hear or see,” “Visual distortions” (β ≥.063; *ps* ≤.002) (Table 2).

### Ayahuasca mental adverse effects relationships with participants’ current mental health

The relationships of post ayahuasca use adverse mental states on participants’ current mental health are presented in Table 3. Five of the eight adverse states included in the model were significantly related to participants’ current mental health. Participants’ reported increases of “Visual distortions” (β =.074; *p* =.004) was related to better current mental health. However, “Little interest or pleasure in doing things,” “Feeling down, depressed, or hopeless,” “Feeling disconnected or alone,” and “Feeling energetically attacked or a harmful connection with a spirit world” (β ≤ -.074; *ps* ≤.026) were related to participants’ worse current mental health. Among the post ayahuasca use adverse states that were negatively related to participants’ current mental health, “Feeling down, depressed, or hopeless” presented the highest effect (β = -.200; *p* <.001).

**Table 3:** Post-ayahuasca use adverse mental states relationships with participants’ current mental health.

	Z	*p*	*β*	*se*	π
**Post-ayahuasca use adverse mental states**					
Feeling nervous, anxious, or on edge	.08	.939	-.002	.025	.950
Little interest or pleasure in doing things	2.77	.006	-.081	.028	.950
Feeling down, depressed, or hopeless	5.22	<.001	-.200	.036	.999
Feeling desconected or alone	2.22	.026	-.074	.030	.940
Difficulty knowing what is real and not real	.377	.706	-.011	.025	.970
Hearing or seeing things that other people do not hear or see	.326	.744	-.009	.028	.940
Visual distortions	2.88	.004	.074	.025	.990
Feeling “energetically attacked” or a harmful connection with a “spirit world”	2.15	.032	-.052	.022	.970

### Exogenous variables indirect relationships with participants’ current mental health

For clarity in the results section, only the main indirect effects results are presented here. The complete results of the exogenous variables indirect effects through those adverse states that showed significant relationships with participants’ current mental health are presented in Table C in S1 Text. While the majorities of the exogenous variables were not significant related with participants’ current mental health through the adverse states, previous depression, spiritual significance and extreme fear were significant related with mental health through the majorities of the adverse stated. Concretely, higher intensity of extreme fear was significant related with lower current mental health through “Little interest or pleasure in doing things” (β = -.018; *p* =.007), “Feeling down, depressed, or hopeless” (β = -.019; *p* =.029), “Feeling desconeted or alone” (β = -.019; *p* =.029), and “Feeling energetically attacked or a harmful connection with a spirit world” (β = -.016; *p* =.033), but it also was positively related with mental health through “Visual distortions” (β =.012; *p* =.006). Contrary to the observed with extreme fear, higher intensity of the participants spiritual significant experiences was positively related with current mental health through the adverse states (Little interest or pleasure in doing things”: β =.006; *p* =.022; “Feeling down, depressed, or hopeless”: β =.016; *p* =.001; “Visual distortions” (β =.006; *p* =.019). Finally previous depression significantly reduces current participants’ mental health through “Feeling down, depressed, or hopeless” (β = -.058; *p* <.001), “Feeling desconeted or alone” (β = -.018; *p* =.035), but it was related with higher mental health through “Visual distortions” (β =.011; *p* =.033) (see Table C in S1 Text).

### Exogenous variables direct relationships with participants’ current mental health

The direct effects of exogenous variables on participants’ current mental health are presented in Table 4. Higher participant-reported ayahuasca use variables were related to better participants’ current mental health (β ≥.097; *ps* <.001), with last year’s use having the strongest relationship with participants’ current mental health (β =.170; *p* <.001). In contrast to the history of ayahuasca use variables, previous mental disorders were related to worse current mental health (β = -.184 and -.240; *p* <.001 for anxiety and depression, respectively). Finally, while participants’ reported intensity of spiritual significance was related to better mental health (β =.101; *p* <.001), the intensity of extreme fear (β = -.054; *p* <.001) was related to worse participants’ current mental health.

**Table 4:** Exogenous variables direct effects on participants’ current Mental Health.

	Z	*p*	*β*	*se*	π
**Exogenous variables direct effects**					
Education	3.43	.001	-.041	.012	.999
Age	.74	.456	.020	.026	.970
Last Year Uses	8.06	<001	.170	.021	.999
Age of Onset	3.82	<.001	.107	.028	.999
Lifetime Uses	3.73	<.001	.097	.026	.999
Anxiety	5.38	<.001	-.184	.034	.999
Depression	7.74	<.001	-.240	.011	.999
Spiritual significance	9.49	<.001	.101	.011	.999
Extreme fear	4.22	<.001	-.054	.013	.999
**Total exogenous variables effects on participants’ current Mental Health**					
Last year use	8.60	<.001	.181	.028	.999
Age of onset	3.03	.002	.097	.035	.999
Lifetime use	3.30	.001	.088	.032	.999
Anxiety	6.28	<.001	-.220	.049	.999
Depression	8.95	<.001	-.330	.044	.999
Spiritual significance	11.36	<.001	.130	.013	.999
Extreme fear	8.96	<.001	-.151	.015	.999

^1^: partially standarized stimates are reported for dichotomical variables.

### Total effects on participants’ current mental health

Finally, to test our main hypothesis, we performed the total effect (the direct effect plus the indirect effect through the post ayahuasca use adverse states) of each of the ayahuasca history of use, clinical, and ayahuasca acute experiences variables. All the ayahuasca history of use variables studied were related to participants’ better current mental health (β ≥.088; *p*s ≤.001). Participants’ reported history of anxiety and depression were related to worse current mental health (β ≤ -.220; *ps* <.001). The participants’ reported intensity of spiritual significance was related to better mental health (β =.130; *p* <.001), while the intensity of extreme fear was related to worse participants’ current mental health (β = -.151; *p* <.001) (Table 4).

## Discussion

In our previous analysis of the database from the Global Ayahuasca Survey (GAS), we found a prevalence of adverse mental states after ayahuasca use in almost 60% of the sample. However, approximately 88% of respondents considered such states as part of a positive process of growth and integration [6]. Further analysis of other variables collected in the GAS revealed improvements in mood disorders such as anxiety and depression [68], as well as reductions in alcohol and drug use [69], enhancements in mental health and wellbeing [70], and changes in life and lifestyle [71]. We have also reported, through qualitative analysis, that ayahuasca experiences can be challenging and that facilitating positive growth may depend on working through those integration challenges [72]. Taking all these data together, it seems necessary to explore deeper into the knowledge of the so-called adverse mental states following ayahuasca use because of their unusual psychopharmacological nature. Such effects, depending on a series of factors, may lead to benefits rather than harms [32,41,45,46].

In this extended analysis of the adverse mental states after ayahuasca use based on data from the Global Ayahuasca Survey (GAS) [6], we have used machine learning and statistical analysis to identify specific relationships between these adverse mental states, participants’ current mental health, and key influencing factors.

From the 5,400 subjects included in the analysis, almost 60% of the sample had used ayahuasca more than 31 times, suggesting a sufficiently reliable sample for carefully studying the potential adverse effects of ayahuasca. Furthermore, half of the sample were women, allowing for an analysis of potential adverse events segregated by gender. The mean age of our sample was 40.9 years, with the initiation of ayahuasca use at 31.1 years and a high educational level. This suggests, from a sociological perspective, that individuals who start and continue using ayahuasca are mature, with their personalities already formed, and that ayahuasca use is not merely about seeking to get high. This aligns with previous personality studies which found that users do not score higher than controls on novelty seeking but do score higher on self-transcendence both in religious [37,38,73,74] and non-religious settings [32]. Additionally, both ethnographic and studies using public health-based indicators conclude that participating in ayahuasca ceremonies is a form of self-care and self-attention in both religious and non-religious users [34,35,75].

Although our primary aim was to analyze the mediational relationship of the adverse mental states reported after ayahuasca use on mental health, this study enabled us to draw a general conclusion about the effect of ayahuasca use on users’ current mental health. As indicated in previously cited studies [32,41,42,76], the results from various analyses performed showed that more ayahuasca use is related to better current mental health in users. Concretely, all the ayahuasca history of use variable were among the more important predictors of current mental health in Random Forest analysis and, despite ayahuasca use was related with higher increases of some adverse mental states, the total effects of these variables in the mediational analysis showed that higher use is related to better participant’s current mental health. Furthermore, the sample SF-12 Mental Health score, (mean 50.16 and standard deviation 9.11), closely matches the expected score for the general population (mean 50, standard deviation 10) [77]. In that sense, although there are subsamples with histories of mood, substance, and drug disorders, our sample is completely normal in terms of mental health. Thus, we find initial evidence of the psychological safety of ayahuasca in general terms, independent of specific cases of adverse consequences of its use. From this finding, we could extrapolate that generally, ayahuasca practices do not pose a risk to public health.

Of the 24 initial predictors of mental health, 19 were identified as the most significant in assessing participants’ current mental health. Alcohol and substance use disorders, along with the adverse post-ayahuasca states of “not being able to stop or control worrying” and “nightmares or disturbing thoughts, feelings, or sensations,” showed little relevance, despite the latter typically being associated with depression, anxiety, and PTSD [78]. This may reflect symptomatic improvements within the sample, though the relatively low prevalence of these disorders (19.7% depressive, 14.2% anxiety, 9.2% substance use, and 10.0% alcohol use disorders) could also explain their diminished importance. Further research is needed to explore potential reductions in sleep disturbances, as effective treatments for nightmares remain critical for mental health populations [79].

Interesting results were also observed when analyzing the relationships between the predictors and the increases of the ayahuasca adverse states. Higher after use increases of “Hearing or seeing things that other people do not hear or see,” “Visual distortions,” and “Feeling ‘energetically attacked’ or a harmful connection with a spirit world” were observed in those participants with a higher frequency of ayahuasca use. More interesting is the result of no significant relationships of the history of ayahuasca use variables with the increases of the majority of the adverse emotional-cognitive states.

On the other side, higher increases of all the adverse effects, except for “Feeling nervous, anxious, or on edge,” was related to having a previous depressive disorder. In that same sense, participants’ previous anxiety disorder was the most significant predictor of experiencing any adverse effect from both the emotional-cognitive and psychotomimetic categories [6], a finding that was also observed in a study with regular religious ayahuasca ceremony attendees [80]. Anxiety disorders have led to psychological adverse states even in laboratory settings [81]. Conversely, this study showed that previous anxiety disorder only significantly increased the two adverse “anxiety-like symptoms” mental states included in the study: “Feeling nervous, anxious, or on edge” and “Difficulty knowing what is real and not real.” Thus, the increment of adverse emotional-cognitive experiences seems to be more strongly related to users’ vulnerabilities rather than to the history of ayahuasca use variables studied. These findings align with current studies that highlight the importance of studying users’ vulnerabilities to better understand the psychedelic experience [45,46,82]. This emphasis on individual vulnerability underscores the complex interplay between pre-existing mental health conditions and the psychoactive effects of ayahuasca, pointing to a nuanced understanding of how such experiences impact different users.

We have found that experiencing ayahuasca ceremonies as spiritually significant increases the psychotomimetic states (“Hearing or seeing things that other people do not hear or see” and “Visual distortions”), but it also reduced the adverse emotional-cognitive states (“Feeling nervous, anxious, or on edge”, “Little interest or pleasure in doing things”, “Feeling down, depressed, or hopeless”, “Feeling disconnected or alone”). So, ayahuasca spiritual significant experiences are related to better current mental health by directly increasing mental health and the psychotomimetic effects, but also by reducing the adverse emotional-cognitive states. In accordance with our results, the relationships of spirituality with better mental health have been reported previously [83]; moreover, psilocybin has been also linked in a dose-dependent way with mystical-type experiences and wellbeing [84]; and finally, mystical significance has been recently related with the beneficial effects of different psychedelic substances [44], or considered a necessary experience for the beneficial effects of these substances [43].

Experiencing extreme fear during the ceremonies was the strongest predictor of all the adverse mental states reported after ayahuasca use, showing the greatest effect among all the studied variables in increasing post-use adverse states. Despite the necessary caution due to our study design, it is interesting to recall that fear has traditionally been linked to anxiety (e.g., [44]), and anxiety has been related to fearfulness (e.g., [85]). Moreover, the complexity of defensive networks is central in emotional processing and response (e.g., [86]). This complexity has been related not only to fear and anxiety but also to depression, as all these states are connected with the functional interplay of top-down and bottom-up processing [87], and these neurological processes have been related to the ayahuasca experience as well [88]. Experiencing extreme fear during ceremonies and prior anxiety disorders, linked to emotional processing, warrant further study to better manage adverse post-ayahuasca mental states and enhance positive outcomes.

Cultural background influences the quality and intensity of adverse mental states after ayahuasca use, with participants from non-traditional contexts reporting higher feelings of disconnection but fewer visual distortions, emphasizing the need for culturally diverse studies given the established cultural differences in how psychedelics affect various domains of mental health [89–91].

Despite 10 post-ayahuasca use adverse mental states being initially included in the analyses, only 5 were significantly related to participants’ current mental health. Four of these adverse states were negatively related to mental health, while the increase in “visual distortion” was related to better participants’ current mental health. The adverse states strongly related to poorer current mental health were all mentioned above as the “depression-like symptoms” adverse states (“Little interest or pleasure in doing things”, “Feeling down, depressed, or hopeless”, and “Feeling disconnected or alone”), while “Feeling nervous, anxious, or on edge”, which was strongly predicted by previous anxiety disorder, was not significantly related to current mental health. Moreover, a previous depressive disorder emerged as one of the strongest predictors of negative impacts on participants’ current mental health among all the adverse states analyzed. This finding appears counterintuitive considering existing evidence on the positive effects of psychedelic therapy for depressive symptoms [92]. From a descriptive point of view, this result emphasizes the importance of caution when ayahuasca users with a history of previous depressive disorders experience after ayahuasca use an increased states of “Feeling down, depressed, or hopeless” and highlights an interesting focus of research in the complexity of the psychedelic experience as has been proposed recently [93]. Finally, another interesting result and future topic of research is the positive relationship of experiencing visual distortion and spiritual significance with better current ayahuasca users’ mental health.

Finally, despite not being a main topic of analysis in this study, since we did not find any notable results related to gender, there is still something to consider regarding gender differences in adverse mental states after ayahuasca use. In our previous classical regression analysis, we found that being female significantly increased the risk of experiencing adverse states [6]. However, in this study, which looks more closely at the potential mediational variables of suffering adverse states, being female was not related to participants’ current mental health. So, even if women suffer more adverse states after ayahuasca use than men, that does not imply a negative consequence for their mental health. To our knowledge, there are no other gender studies on ayahuasca’s adverse effects, and even in a recent systematic review on the topic, results were not segregated by gender [94]. Other studies focused on studying adverse effects of psychedelics neither analyze their results by gender nor from a gender perspective [45,46,82,95]. In a study with a sample of 380 regular Spanish ayahuasca users (47% women), there were no differences between genders in the GHQ-12 questionnaire, which measures mental health, while in the general population men have 10 points better mental health than women [34]. There are two hypotheses: that women with better mental health are the ones who attend ayahuasca ceremonies, or that participating in ayahuasca ceremonies improves mental health of participants, especially women.

In previous results from the GAS, gender disappeared as a predictor of improvements in mental health and wellbeing [42], and life and lifestyle changes [71]. Moreover, some field researches have shown how women create protective strategies when they get involved in the use of psychoactive plants, by means of enhancing social cohesion through women circles and other self-care strategies [75,96]. An alternative explanation for the good outcomes on women’s mental health despite their greater presence of potential adverse effects may be the social protective factors that they deploy. This may also explain how mental health adverse states were negatively associated with consumption in religious settings, as we found in our previous study [6]. Perhaps the explanation is not based on the religious context but on the fact that the ayahuasca use was conducted in the context of a cohesive group where there is social support among members. When designing different contexts of use, we did not create a category referred to taking ayahuasca in a cohesive group, something that future studies should consider. This aspect is of special interest in this context of ‘psychedelic renaissance’, where psychedelics are becoming medicalized while communal use is prosecuted [97] in a world suffering a global mental health pandemic where the loss of social bonds and self-perception of loneliness are among its main causes [98].

As this study is a second analysis of the GAS sample relating to adverse mental states, it is affected by the same limitations our previous one. The study design prevents us from establishing causality, and the retrospective evaluations of predictors and adverse states, as well as the self-selection bias, are all limitations that must be considered [6]. Specifically, first, recall bias may affect participants’ memories of past experiences, especially in cases where ayahuasca use occurred months or years prior. Additionally, selective reporting bias could influence participants to emphasize positive or negative experiences based on personal beliefs or expectations about ayahuasca. The survey’s voluntary and self-selected nature may also attract individuals with particularly strong opinions or unusual experiences, potentially skewing the results. Finally, cultural and contextual variations among participants from different regions may introduce variability that affects the interpretation of findings across diverse populations. Moreover, lower frequencies of lifetime depression and substance use disorders were found in the participants excluded compared with those included. Despite this, the sample size and its global representativeness make the study results interesting to explore possible relationships between the studied variables. Spiritual significance and extreme fear were treated as predictors. Despite the covariance between exogenous variables in the mediational test being fixed to the dataset values, which allows for accounting for the relationships between exogenous variables, both experiences should be treated in future studies as mediational ones using intensity measures [99]. Finally, an interesting result, which should not necessarily be considered a limitation, was that despite the high explained variance for participants’ current mental health (30.0%), the explained variance for the increment of the adverse mental states was lower (ranging from 6.0% to 20.6% for ‘Visual distortions’ and ‘Difficulty knowing what is real and what is not’). Thus, other predictors not included in our study must be considered in future studies to better understand the so-called post-ayahuasca use adverse mental states.

Despite the limitations, some conclusions can be drawn from this study’s results. In a sample of frequent ayahuasca users, although its use is associated with some negative reactions, higher usage is related to better mental health. The majority of the so-called ayahuasca adverse states did not have a negative impact on users’ current mental health. However, adverse depressive-like symptoms experienced after ayahuasca use seem to be related to poorer mental health, while experiencing visual distortions has a positive effect on users’ current mental health. Therefore, it may be necessary to reconceptualize what are considered adverse mental states after ayahuasca use, since some effects that have traditionally been defined as “adverse” actually lead to positive outcomes, as is the case with the ones just referred to, and other, as those adverse anxiety-like symptoms could be related with user symptoms improvement. Moreover, our results underline the importance of studying individual vulnerabilities when analyzing the beneficial or harmful consequences of ayahuasca adverse reactions, as spiritual significance, extreme fear, and previous mental disorders were observed to be both beneficial and harmful factors. Finally, it is warranted to explore the roles of social bonding in the positive mental health outcomes of participants in ayahuasca ceremonies as a possible determinant factor in improving the mental health of participants [100].

*Practical, professional, and policy implications of our observed mediational effects*. Our analysis identified distinct mediational effects of post-ayahuasca mental states that impact participants’ current mental health, revealing both detrimental and beneficial influences shaped by contextual and individual factors. Experiences of extreme fear during ceremonies were associated with a higher likelihood of all adverse post-ceremony mental states, which in turn correlated with poorer mental health outcomes. Ayahuasca experience are modulated by non-pharmacological factors [42,76] like set (personal disposition) and setting (context). One way to mitigate potential experiences of fear is to work beforehand with the person who will take ayahuasca, offering them appropriate information about both the positive and negative effects of the plant, reassuring them they will be cared for, and instilling trust. It is essential that those conducting ceremonies spend time before the ceremony speaking with attendees, both individually and as a group. Maintaining a space that is considered safe can also help prevent experiences of fear.

Specific adverse states, such as emotional disconnection, sensations of feeling “energetically attacked” or connected to a harmful “spirit world,” and feelings of hopelessness or depression, were linked to significant declines in mental health, particularly among participants in non-traditional settings and those with pre-existing depressive disorders. Again, it is crucial that guides spend time talking to attendees. In our experience, what are referred to as “spiritual attacks” can be highly threatening, and people may end up traumatized. For shamanism, as well as for some ayahuasca churches, healing occurs in the spiritual realm, where friendly forces battle hostile ones. This explains the occasional experience of feeling spiritually attacked. Guides must ensure attendees feel protected during ceremonies. Regardless of whether one believes in this ayahuasca-based conception of healing, it is essential that guides communicate confidence that they, not the attendees, are the ones dealing with hostile forces in the spiritual realm. If someone feels spiritually attacked after the ceremony, it is necessary to initiate an integration process to help them reframe their experience [73,101]. Guides also need to pay attention to individuals who show signs of depression and provide them with special care, as these individuals may be at a higher risk of worsening mental health outcomes. Paradoxically, clinical trials have found an antidepressant effect of ayahuasca [92]. This discrepancy with our results may be due to the fact that, in clinical trials, patients receive extensive attention. Thus, providing additional attention to individuals with a history of depression could potentially prevent negative outcomes.

On the other hand, certain adverse effects were associated with improved mental health. Visual distortions, for example, positively correlated with better current mental health, especially in participants who reported frequent ayahuasca use. Additionally, the spiritual significance of the experience acted as a protective factor, reducing adverse emotional effects such as anxiety and disconnection while fostering overall mental health benefits. Knowing these positive effects of visual distortions and spiritual significance, guides can emphasize these aspects in pre-experience interviews with participants, helping them understand and appreciate the spiritual importance of the experience.

Contextual and personal background variables further influenced these outcomes: participants in non-traditional ayahuasca settings and those from non-traditional countries experienced higher rates of adverse effects (e.g., emotional disconnection and difficulties discerning reality) that were detrimental to their mental health. This phenomenon may be related to the preparation of the guides, who, given their cultural background in traditional ayahuasca-using contexts and countries, are often highly experienced (they may be *curanderos*, *taitas*, *onayas*, *mestres*, *maestros*, *pajés*, etc.). Therefore, it is important for those seeking ayahuasca ceremonies to do so in contexts and with people where there is a long-standing tradition of use, where negative experiences are less likely to occur.

Last, in terms of policy implications, ayahuasca ceremonial practices have spread across the planet [3], and the participation in the GAS of people from more than 50 countries is further evidence of this. A recent report by the NGO ICEERS estimated that around 4 million people have taken ayahuasca all over the world [102], and it does not appear that this phenomenon is decreasing; on the contrary, it seems to be growing. The aim of this paper is not to discuss the causes of this phenomenon. What is evident is that the phenomenon exists even in the face of punitive measures, as in the cases of France, Italy, Russia, and China, where ayahuasca use is specifically targeted and penalized. Although DMT is controlled according to the United Nations 1971 Convention on Psychotropic Substances, neither ayahuasca nor any preparation made from plants containing DMT is controlled [103]. In contrast to punitive countries, there are others like Peru, Colombia, and Brazil where certain levels of protection are in place. We have seen that guides and the context in which the experience takes place are very important for the mental health outcomes of participants in ceremonies. The more persecuted the ceremonies are, the more likely it is that safety criteria will not be met. Recently, the Government of Catalonia published a safety guide for ayahuasca facilitators created by the ICEERS Foundation [104]. This is a measure that other regions and countries could adopt. But perhaps the most important step would be to create a regulatory framework that establishes a set of criteria and safety measures that must be met by centers and the people who work in them. In Spain, the Netherlands, and other countries, initiatives are underway in which guides are forming associations that establish safety criteria and ethical codes that require compliance among their members. For administrations to guarantee the legal safety of these associations is another way to ensure the safety of participants. Obviously, these measures should only be implemented outside indigenous contexts, as indigenous communities already have their own forms of self-regulation. Allowing the political and legal context for good practices to occur is the best way to prevent negative consequences of participation in ayahuasca ceremonies and enhance the positive ones.

## Supporting information

S1 Text Supporting Tables. (DOCX)
