# Supplementary material for: A new insight into ayahuasca’s adverse effects: Reanalysis and perspectives on its mediating role in mental health from the Global Ayahuasca Survey (GAS)
Source: PLOS Ment Health. 2025 Apr 30;2(4):e0000097. doi: 10.1371/journal.pmen.0000097 (PMC12798354; doi:10.1371/journal.pmen.0000097)
Supplement: S1 Text — (DOCX) [file pmen.0000097.s001.docx]

| **Table A in S1 text: Recursive feature selection (bootstrapped method by 50 repetitions)** | | |
| --- | --- | --- |
| Variables | R^2^ | RMSE |
| Age (E) | .026 | 10.05 |
| Age of onset (E) | .065 | 9.00 |
| Last year uses (E) | .087 | 8.83 |
| Extreme fear (Ad) | .112 | 8.68 |
| Spiritual significance (Ac) | .136 | 8.54 |
| Education (E) | .124 | 8.71 |
| Depression (E) | .153 | 8.51 |
| Lifetime uses (E) | .176 | 8.37 |
| Feeling disconnected or alone (Ad) | .191 | 8.31 |
| Sex (E) | .211 | 8.18 |
| Visual distortions (Ad) | .222 | 8.11 |
| Anxiety (E) | .224 | 8.11 |
| Hearing or seeing things that other people do not hear or see (Ad) | .231 | 8.07 |
| Feeling down, depressed, or hopeless (Ad) | .238 | 8.03 |
| Feeling “energetically attacked” or a harmful connection with a “spirit world” (Ad) | .240 | 8.02 |
| Feeling nervous, anxious, or on edge (Ad) | .246 | 7.99 |
| Traditional/no Traditional Context (E) | .251 | 7.96 |
| Little interest or pleasure in doing things (Ad) | .252 | 7.95 |
| Traditional/no Traditional Country (E) | .254 | 7.94 |
| Difficulty knowing what is real and not real (Ad) | .256 | 7.92 |
| Nightmares, or disturbing thoughts, feeling, or sensations (Ad) | .256 | 7.93 |
| Not being able to stop or control worrying (Ad) | .258 | 7.92 |
| Drug use disorder (E) | .259 | 7.91 |
| Alcohol use disorder* (E) | .257 | 7.92 |
| E: Exogenous variable; Ad: adverse mental states; Ac: acute effects, * : Variable excluded by the procedure; Higlightened in gray the last predictor included in the following analyses. | | |

| **Table B in S1 text: Variables scaled importance score from Random Forest mode** | |
| --- | --- |
| Variables | Importance Score |
| Age | 100.00 |
| Last year uses | 93.48 |
| Lifetime uses | 79.46 |
| Age of onset | 76.70 |
| Feeling disconnected or alone | 61.56 |
| Extreme fear | 60.44 |
| Spiritual significance | 60.08 |
| Feeling down, depressed, or hopeless | 52.14 |
| Little interest or pleasure in doing things | 37.51 |
| Education | 35.73 |
| Feeling nervous, anxious, or on edge | 28.35 |
| Depression | 20.98 |
| Anxiety | 13.70 |
| Feeling “energetically attacked” or a harmful connection with a “spirit world” | 12.95 |
| Visual distortions | 7.95 |
| Difficulty knowing what is real and not real | 6.94 |
| Hearing or seeing things that other people do not hear or see | 6.23 |
| Traditional/no traditional country | 4.27 |
| Traditional/no traditional context | 2.79 |
| Sex | 0.00 |
|  | |

| **Table C in S1 text: Exogenous variables indirect relationships with participants current mental health status** | | | | | |
| --- | --- | --- | --- | --- | --- |
|  | **Z** | ***p*** | ***β*** | ***se*** | **π** |
| **Through “Little interest or pleasure in doing things”** | | | | | |
| Education | 1.67 | .095 | .004 | .002 | .960 |
| Age | 1.86 | .063 | .008 | .004 | .970 |
| Last year uses | .332 | .740 | -.001 | .003 | .990 |
| Age of onset | 1.01 | .312 | -.004 | .003 | .980 |
| Lifetime uses | .037 | .971 | -.001 | .003 | .980 |
| Anxiety | 1.24 | .213 | -.007 | .006 | .990 |
| Depression | 1.72 | .085 | -.009 | .005 | .980 |
| Traditional/no traditional context | .574 | .566 | -.002 | .004 | .970 |
| Traditional/no traditional country | .571 | .568 | .002 | .003 | .950 |
| Spiritual significance | 2.29 | .022 | .006 | .004 | .960 |
| Extreme fear | 2.70 | .007 | -.018 | .007 | .950 |
| **Through “Feeling down, depressed, or hopeless”** | | | | | |
| Education | .90 | .365 | .004 | .004 | .980 |
| Age | 2.98 | .003 | .030 | .010 | .999 |
| Last year uses | .98 | .326 | .007 | .007 | .999 |
| Age of onset | 1.91 | .056 | -.018 | .009 | .999 |
| Lifetime uses | 2.11 | .034 | -.019 | .009 | .999 |
| Anxiety | 1.27 | .260 | -.013 | .011 | .970 |
| Depression | 4.01 | <.001 | -.058 | .014 | .999 |
| Traditional/no traditional context | 2.62 | .009 | -.026 | .010 | .960 |
| Traditional/no traditional country | 1.47 | .142 | -.014 | .010 | .980 |
| Spiritual significance | 3.41 | .001 | .016 | .005 | .999 |
| Extreme fear | 2.19 | .029 | -.019 | .009 | .999 |
| **Through “Feeling disconnected or alone”** | | | | | |
| Education | 1.75 | .079 | .004 | .002 | .940 |
| Age | 1.98 | .048 | .010 | .005 | .940 |
| Last year uses | .78 | .435 | .002 | .002 | .970 |
| Age of onset | 1.45 | .147 | -.005 | .003 | .960 |
| Lifetime uses | .16 | .873 | -.001 | .003 | .980 |
| Anxiety | 1.42 | .156 | -.007 | .005 | .950 |
| Depression | 2.11 | .035 | -.018 | .009 | .940 |
| Traditional/no traditional context | 2.04 | .041 | -.014 | .007 | .960 |
| Traditional/no traditional country | 1.82 | .069 | -.010 | .005 | .950 |
| Spiritual significance | 1.82 | .068 | .004 | .002 | .950 |
| Extreme fear | 2.19 | .029 | -.019 | .009 | .940 |
| **Through “Visual distortions”** | | | | | |
| Education | 2.02 | .044 | -.004 | .002 | .999 |
| Age | 2.33 | .020 | -.009 | .004 | .990 |
| Last year uses | .342 | .732 | -.001 | .002 | .970 |
| Age of onset | 2.24 | .025 | .008 | .004 | .990 |
| Lifetime uses | 2.35 | .019 | .011 | .005 | .990 |
| Anxiety | .102 | .919 | .001 | .004 | .990 |
| Depression | 2.14 | .033 | .011 | .005 | .990 |
| Traditional/no traditional context | 1.98 | .048 | .008 | .004 | .990 |
| Traditional/no traditional country | 2.41 | .016 | -.014 | .006 | .970 |
| Spiritual significance | 2.35 | .019 | .006 | .002 | .990 |
| Extreme fear | 2.75 | .006 | .012 | .004 | .990 |
| **Through “Feeling “energetically attacked” or a harmful connection with a “spirit world””** | | | | | |
| Education | 1.28 | .199 | .002 | .001 | .990 |
| Age | 2.02 | .043 | .010 | .005 | .970 |
| Last year uses | 1.60 | .109 | .004 | .003 | .999 |
| Age of onset | 1.78 | .075 | -.006 | .003 | .980 |
| Lifetime uses | 2.00 | .045 | -.012 | .006 | .970 |
| Anxiety | 1.40 | .162 | -.006 | .004 | .990 |
| Depression | 1.94 | .052 | -.012 | .006 | .970 |
| Traditional/no traditional context | 1.90 | .057 | -.009 | .005 | .980 |
| Traditional/no traditional country | 1.93 | .053 | .010 | .005 | .990 |
| Spiritual significance | .945 | .344 | -.001 | .001 | .999 |
| Extreme fear | 2.13 | .033 | -.016 | .007 | .970 |
| ^1^: partially standarized estimates are reported for dichotomical variables | | | | | |
